# Adaptive Color Polymorphism and Unusually High Local Genetic Diversity in the Side-Blotched Lizard, *Uta stansburiana*

**DOI:** 10.1371/journal.pone.0047694

**Published:** 2012-10-25

**Authors:** Steven Micheletti, Eliseo Parra, Eric J. Routman

**Affiliations:** Department of Biology, San Francisco State University, San Francisco, California, United States of America; Institut de Biologia Evolutiva - Universitat Pompeu Fabra, Spain

## Abstract

Recently, studies of adaptive color variation have become popular as models for examining the genetics of natural selection. We examined color pattern polymorphism and genetic variation in a population of side-blotched lizards (*Uta stansburiana*) that is found in habitats with both dark (lava) and light colored (granite) substrates. We conducted a limited experiment for adult phenotypic plasticity in laboratory conditions. We recorded both substrate and lizard color patterns in the field to determine whether lizards tended to match their substrate. Finally we examined genetic variation in a gene (*melanocortin 1 receptor*) that has been shown to affect lizard color in other species and in a presumably neutral gene (mitochondrial *cytochrome b*). Populations were sampled in the immediate area of the lava flows as well as from a more distant site to examine the role of population structure. Our captive *Uta* did not change color to match their background. We show that side-blotched lizards tend to match the substrate on which it was caught in the field and that variation in the *melanocortin 1 receptor* gene does not correlate well with color pattern in this population. Perhaps the most remarkable result is that this population of side-blotched lizards shows extremely high levels of variation at both genetic markers, in the sense of allele numbers, with relatively low levels of between-allele sequence variation. Genetic variation across this small region was as great or greater than that seen in samples of pelagic fish species collected worldwide. Statistical analysis of genetic variation suggests rapid population expansion may be responsible for the high levels of variation.

## Introduction

Recently there has been increased focus on the genetic basis of color variation in animals because of the potential for color to be influenced by natural selection for background matching, thermoregulation, and sexual selection, e.g. [1]–[4]. Reptile coloration has long been studied as an example of adaptive evolution [5]–[6]. Although the color of reptile individuals can sometimes vary due to social signaling, stress, or active camouflaging [7], color polymorphism presents an opportunity to study the evolution of phenotypic diversity [5]–[6] as long as the polymorphism is not due strictly to phenotypic plasticity. Diurnal reptiles, especially those residing in the open desert, experience intense selection for substrate matching [8], as dorsal crypsis allows individuals to avoid being detected by highly visual predators [9]–[10]. Furthermore, studies have demonstrated that darker animals can heat faster and obtain higher body temperatures than lighter animals [11]–[12] and light coloration may serve to also slow or limit heat gain [13].

**Figure 1:**
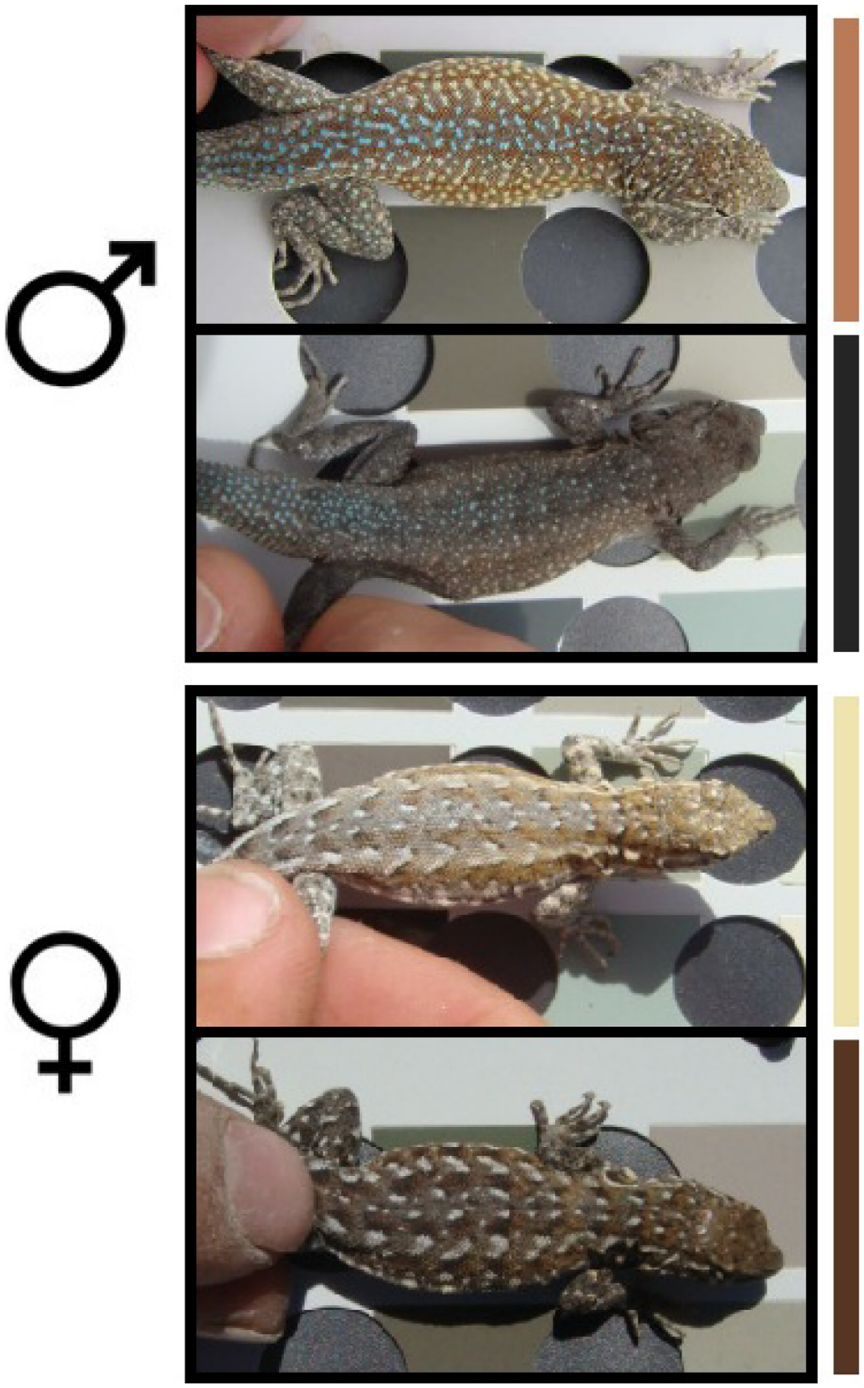
Dorsal Color Variation in *Uta stansburiana*. Male and female *U. stansburiana* collected from granite sands (top picture) and lava (bottom picture). Bars to the right of the photographs represent the color call from the Munsell Color chart (see text).

Dorsal color varies among individuals of common side-blotched lizards (*Uta stansburiana)* that reside in the Cima Volcanic Field in San Bernardino County, California (Figure 1). This volcanic field, also known as Cinder Cones National Natural Landmark in the Mojave National Preserve, consists of dark basalt rocks surrounded by lighter granite sands. In *U. stansburiana*, the derived “melanic” phenotype is darker than the ancestral type and is found in individuals that occupy the darker basalt substrate. If the phenotypic variation in this population is not due to individual phenotypic plasticity, this species can be used to understand the balance between gene flow and adaptive evolution in reptiles. Depending on the strength of natural selection and rates of migration, gene flow may obstruct local adaptation by homogenizing populations subject to different selection pressures [14]–[15]. Alternatively, strong disruptive selection may overwhelm even substantial gene flow and lead to population differentiation [16].

Similar systems have been investigated by Rosenblum and colleagues [17] in the Carrizozo Lava Flow, a very young (∼5000 years old) lava flow in New Mexico [18]. Several species of desert lizards show a light/dark polymorphism in which dorsal color matches the habitat type on which they reside [19]. This finding suggests that natural selection is strong enough to produce a consistent pattern among species despite the fact that the species differ in population structure, historical demography, and ecology. The Cima Lava Field differs from the lava studied by [17] in that it consists of a series of much older lava outcrops (between 7.6 million years old and 10,000 years old) that vary in size and age and are separated by a mixture of basalt rocks and granite sands [20]–[21]. The volcanic field includes 52 volcanic cinder cone vents, and extensive basaltic lava flows that cover more than 150 square kilometers of the Mojave Desert [21].

We examined gene flow and demography by comparing mtDNA sequence among color morphs and among geographic localities. Finally, we conducted a preliminary examination of the genetic basis of the color polymorphism by surveying genetic variation in a candidate locus, melanocortin 1 receptor (*Mc1r*), that is known to affect color pattern in other species of vertebrates [3] [1].

## Methods

### Ethics Statement

This study was carried out in strict accordance with the recommendations in the Guide for the Care and Use of Laboratory Animals of the National Institutes of Health. The protocol was approved by the Institutional Animal Care and Use Committee of San Francisco State University (Protocol Number: A10-002).

### Sampling

**Figure 2:**
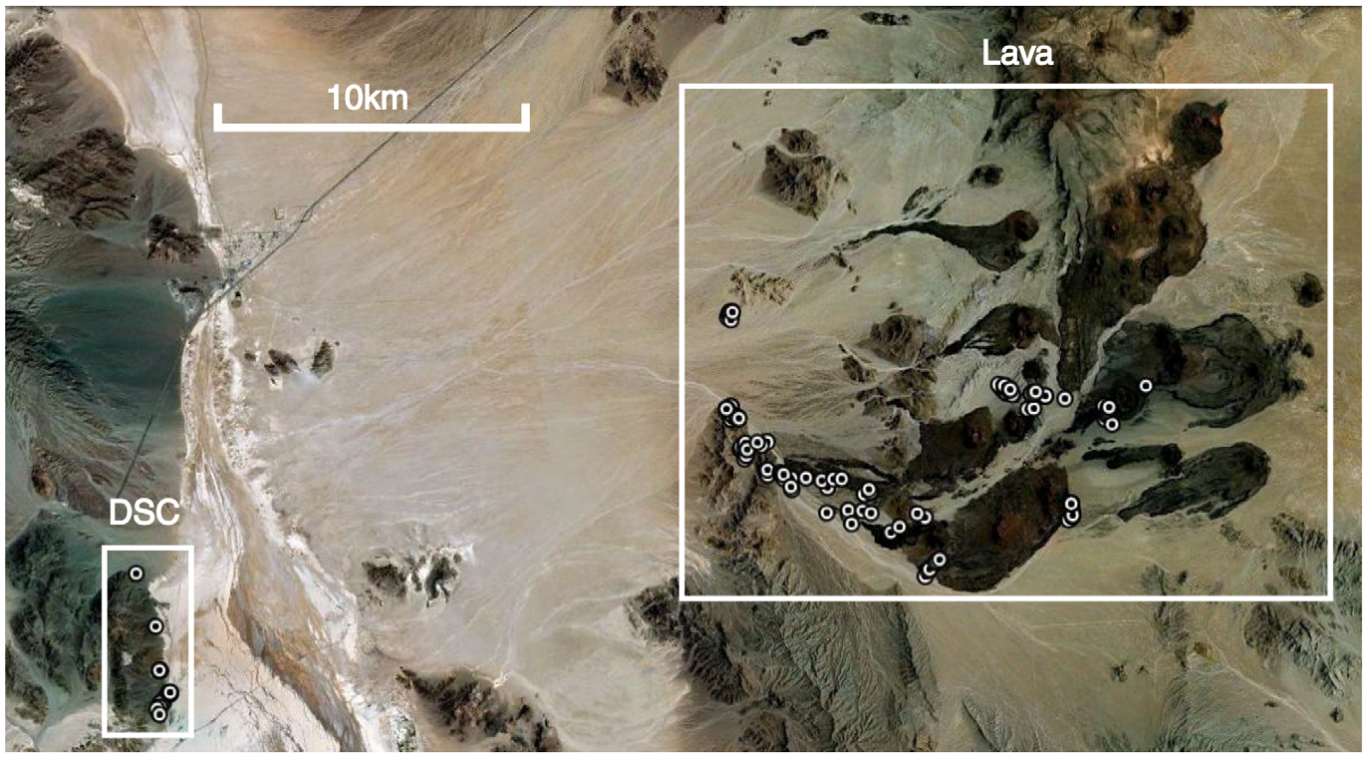
Study Location. Satellite imagery of the Desert Studies Center (DSC), the Cinder Lava Field (Lava) and the soda lake separating the two. Circles indicate collection sites of individual lizards, although at this geographic scale many symbols overlap.

We collected samples from two geographic sites: The Cima Volcanic Field and adjacent habitats (N = 110), and the Desert Studies Center (N = 17) (Table S1; Figure 2). The Cima Volcanic Field was selected as the main study site due to a large variation in substrate color over a small distance. The Desert Studies Center is approximately 23 kilometers west of the Volcanic Field and lacks volcanic rock. The Desert Studies Center site is separated from the Cima Volcanic Field by a massive soda lake consisting of alkaline evaporites, sodium carbonate, and sodium bicarbonate [22]. This predominantly dry lake is uninhabitable and potentially acts as a barrier between sites, forcing possible migrants to travel much farther between sites than the straight line distance of approximately 23 km that separates the two areas.

Lizards were captured using slip-knot nooses and 0.5 cm of tail tip was removed and preserved in 95% ethanol for DNA analysis. We measured the most prominent dorsal color of each lizard and the substrate of which it was found with a Munsell Color Geological Rock-Color Chart (Munsell, North Brunswick, NJ). The Geological Color Chart was selected to measure dorsal color since lizards rely on substrate (rock) matching for predator avoidance [8]. The chart consists of a variety of color chips that classify different colors using three color dimensions: hue, value (lightness), and chroma (color purity). We measured dorsal temperature with an infrared thermometer, and lizards were photographed against the substrate on which they were first discovered as well as against the Geological Color Chart. We determined colors at the time of capture and reconfirmed scores later using the photographs. Lizard color was classified into two categories, light and dark, based on their relative color.

### Test for Phenotypic Plasticity

Twelve individuals were collected and maintained in captivity to test whether dorsal color is a plastic trait in these populations. This sample consisted of 6 light, and 6 dark individuals. Lizards were reared in separate 10-gallon glass aquaria. Aquaria were supplied with white sand, a granite rock for basking, and a granite rock refuge. This substrate best matched an extreme light habitat, like that of the granite sands in the Mojave National Preserve. Lizards were fed crickets *ad libitum* with a 12 hour light/dark schedule. Heat lamps were used during the day to maintain a temperature of 25–31°C and allow behavioral thermoregulation. We recorded dorsal colors with the Munsell Color Geological Rock-Color Chart every week for 52 weeks to measure any color change. Since color is thought to be affected by temperature in most lizard species (9 Norris, 1965) each lizard was measured at four temperature intervals: inactive (16.5–17.5°C), room (20 – 21°C), warm (25.5 – 28.5°C), and basking (30.5 – 32°C).

### DNA Extraction, Amplification and Sequencing

We extracted DNA from alcohol preserved tail tissue using a DNEASY extraction kit (QIAGEN Inc. Valencia, CA). Following extraction, the template DNA was amplified using Polymerase Chain Reaction (PCR) for two mitochondrial loci: NADH dehydrogenase subunit 4 (*ND4*) and cytochrome-B (*cytb*), and one nuclear locus, melanocortin 1 receptor (*Mc1r*) (Tables S2 and S3). We chose to use mitochondrial markers for investigation of population structure due to their high sensitivity to population subdivision because of female inheritance and effective haploidy. *Mc1r* was selected because it is a key switch in a signal transduction pathway in melanin-producing cells [23] and has been implicated in intraspecific color variation in birds, mammals, and reptiles, e.g. [3] [24]–[25]. *Mc1r* polymorphisms have been found to be strongly associated with color differences in some desert squamates [3] making it a candidate gene to investigate in *U. stansburiana* color morphs.

We purified successfully amplified PCR product with Exonuclease I (EXO), and Thermosensitive Alkaline Phosphatase (FastAP; Fisher Scientific, Houston, TX). We sequenced purified product in both directions using Elim Biopharm’s DNA sequencing services, which use ABI 3730xl Sequencers (Applied Biosystems, Carlsbad, CA). Sequencing primers (Table S4) were designed from initial sequence data. Individuals with poor sequence quality, unique occurrence of heterozygosity, or unique sequences were re-sequenced. For the autosomal *Mc1r*, double peaks of approximately equal height present in sequence from both directions were scored as heterozygous. We inferred haplotype phases with the program PHASE 2.1.1 [26].

Sequence data were aligned and edited using the computer program Sequencher 3.1.1 (Gene Codes Corporation, Ann Arbor, MI). Sequences were confirmed to be *U. stansburiana* product by using the BlastN program at the NCBI website (http://blast.ncbi.nlm.nih.gov/Blast.cgi). We used MacClade 4.08a [27] to identify unique haplotypes. All sequences obtained in this study have been deposited in Genbank (cytb: JX481355 - JX481481, nd4: JX481482 - JX481608, mc1r: JX481609 - JX481734)).

### Phylogenetic Analysis

Haplotype trees for both mitochondrial and nuclear sequence were created using MrBayes 3.1.2 [28]. The substitution model GTR + Γ was used for all datasets, because overparameterization has been shown to have much less effect on Bayesian phylogenetic analysis than underparameterization [29]. Priors were the default uninformative priors for MrBayes. Two runs of 4 chains each were conducted for each gene. Each run was 20 million generations, with sampling at every 1000 generations, with 25% of samples discarded as burnin. In every case, run length was sufficient to ensure that the average standard deviation of split frequencies were <0.01 well before the end of the run. Nodes with a posterior probability >0.90 are considered well supported [30]. If Bayesian trees had little statistical resolution, the final tree was drawn using neighbor-joining with uncorrected distances and nodes with Bayesian posteriors >90% indicated on the neighbor joining tree.

### Population Genetic Analysis

Population genetic analyses were performed using Arlequin 3.5 [31]. For these analyses we defined three different sets of populations. First, analyses were run with all samples defined as a single population (N = 127). Second, we defined another contrast consisting of two populations based on collection site: Cima Lava Field and adjacent habitats (N = 110) vs. Desert Studies Center (N = 17). These two localities are separated by the uninhabitable soda lake which potentially splits the sample into two populations. Finally, we divided the samples into two populations based on whether an individual’s dorsum was light (N = 61), or dark (N = 66). Because color is thought to be an adaptive trait for predator avoidance, disruptive selection has the potential to subdivide a population. This may occur for only those loci affecting the trait under selection and loci in linkage disequilibrium with the selected gene, while unlinked genes show panmixia. However, if disruptive selection has caused a reduction in gene flow between phenotypes, other genes may show evidence of population subdivision.

For each defined group, we used pairwise mismatch distribution to test for recent population size expansion [32]. Statistical significance was tested using raggedness index (*r*) [33] and sum of squared deviation test [32]. We estimated per locus diversity as haplotype number and haplotype diversity (*h*, [34] for all populations and putative subpopulations. Nucleotide diversity was estimated with Watterson’s θ (θ, [35] and Nei’s θ (π, [36]. We calculated Tajima’s D [37] to test for departures from neutral expectations.

Analyses of molecular variance (AMOVA; [38] were conducted in Arlequin using the different locality-defined or color-defined groups. FST and ΦST from AMOVA were estimated by using allele frequencies only (FST) or by computing distance matrices based on pairwise differences (ΦST). Statistical significance was estimated using randomization tests with 1000 random permutations. Finally Jost’s D value (D_j_, [39] was calculated using the program Genodive 2.0B20 [40] as an estimator of the degree of differentiation between populations. Jost’s D is better than FST or ΦST at indicating population differentiation when haplotype diversity within populations is high [39].

## Results

### Substrate Matching and Adult Phenotypic Plasticity

Lizard color data is displayed in Table S5. Of 127 sampled individuals, 92% matched the color class of the substrate on which they were collected, suggesting a strong correlation between habitat type and dorsal color. Based on the Munsell Geological Color Chart, dark males have a primary dorsal color of Dark Olive Black and dark females are Olive Gray; light males are associated with Dark Yellowish Brown whereas light females are associated with Light Olive Gray. In both light and dark lizards, the primary dorsal colors in males are darker than females, suggesting a primary dorsal color sexual dimorphism. Only 26 of 127 lizards had color scores that could be considered intermediate between light and dark. Classifying lizards as light, medium, and dark did not affect the analyses that follow (data not shown).

Our tests for phenotypic plasticity due to temperature suggest that *U. stansburiana* do experience plasticity due to temperature. Lizards tend to be darker when cool. These results are compatible with other studies of lizard coloration [11] [41] [12]. The darkening response triggered by lower temperatures varied in intensity for different color morphs. We detected negligible color change in darker (lava flow) lizards when measured at different temperatures. However, light colored lizards were considerably darker when cold. Because all of the lizards scored in native habitats were warm and active, phenotypic plasticity due to temperature does not explain the differences between dark and light lizards in the field.

**Figure 3:**
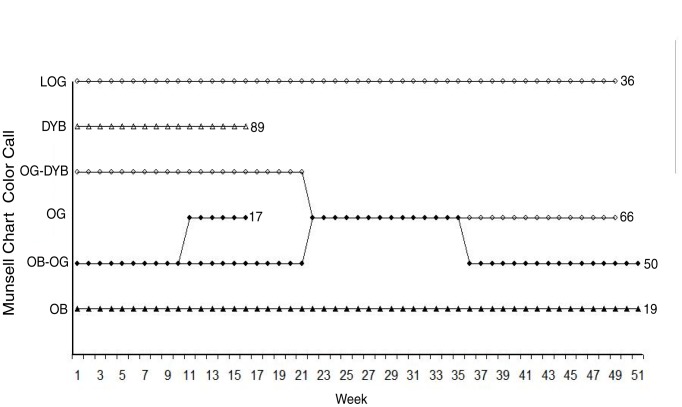
Phenotypic Plasticity of Captive Lizards. Comparison of lizards that were captive for at least 15 weeks. Colors are based on the Munsell Geological Rock-Color Chart, ranging from lighter (top) to darker (bottom): light olive gray (LOG), dark yellow brown (DYB), olive gray (OG), and olive black (OB). Light lizards have open symbols; dark lizards have filled in symbols. Males are indicated with triangles; females are indicated with circles. Numbers at the ends of lines indicate individual lizards.

Of the 12 captive lizards housed on light substrate, only 6 lived 15 weeks or longer. All six short-lived lizards and three of the six longer-lived lizards showed no changes in dorsal color. Three of the 6 lizards that lived 15 weeks or longer showed variation in dorsal color, but this variation did not appear adaptive (Figure 3). Individual 66 surprisingly darkened in dorsal color after 21 weeks of being captive on a light substrate. Individual 50 showed a significant amount of dorsal lightening after shedding, yet darkened again several weeks later. Individual 17 lightened after shedding yet died five weeks later, making it uncertain whether the color change was permanent or not.

To summarize, only 4 of the 12 lizards changed color, and only one became slightly lighter and stayed lighter on the extremely light substrate. Overall, there is no trend towards a color change to match substrate, certainly not in time scales that would be relevant to predator avoidance. This suggests that cryptic coloration in this population of *Uta stansburiana* is probably not due to phenotypic plasticity.

### Phylogenetic Analysis

**Figure 4:**
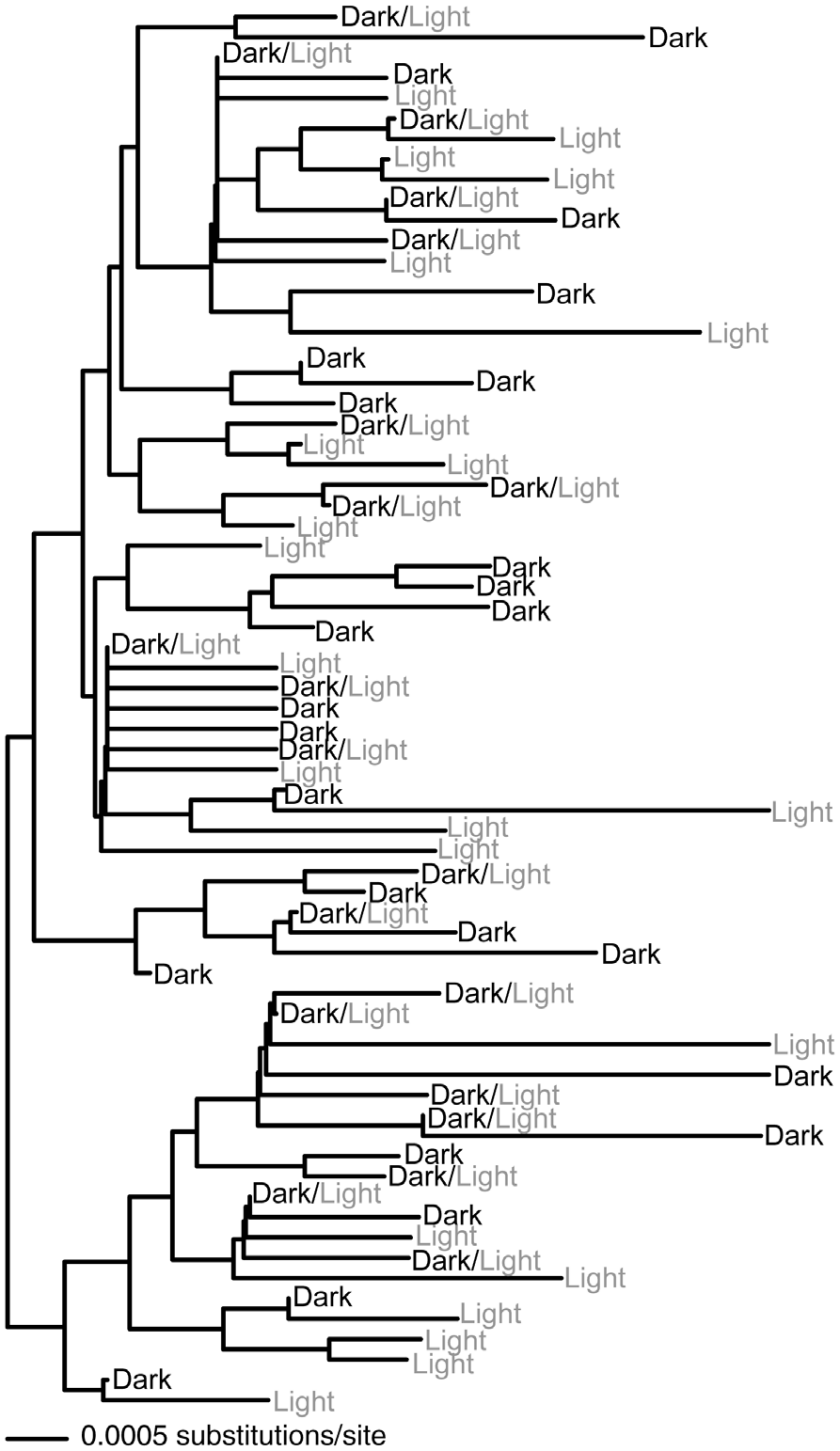
*Mc1r* Phylogeny. *Mc1r* haplotype neighbor joining tree with the 66 identified haplotypes**.** Haplotypes are associated with lizards whose dorsal coloration is either light, dark, or both (dark/light). Bayesian posterior probabilities (from MrBayes) greater than 90 are displayed on branches.

**Figure 5:**
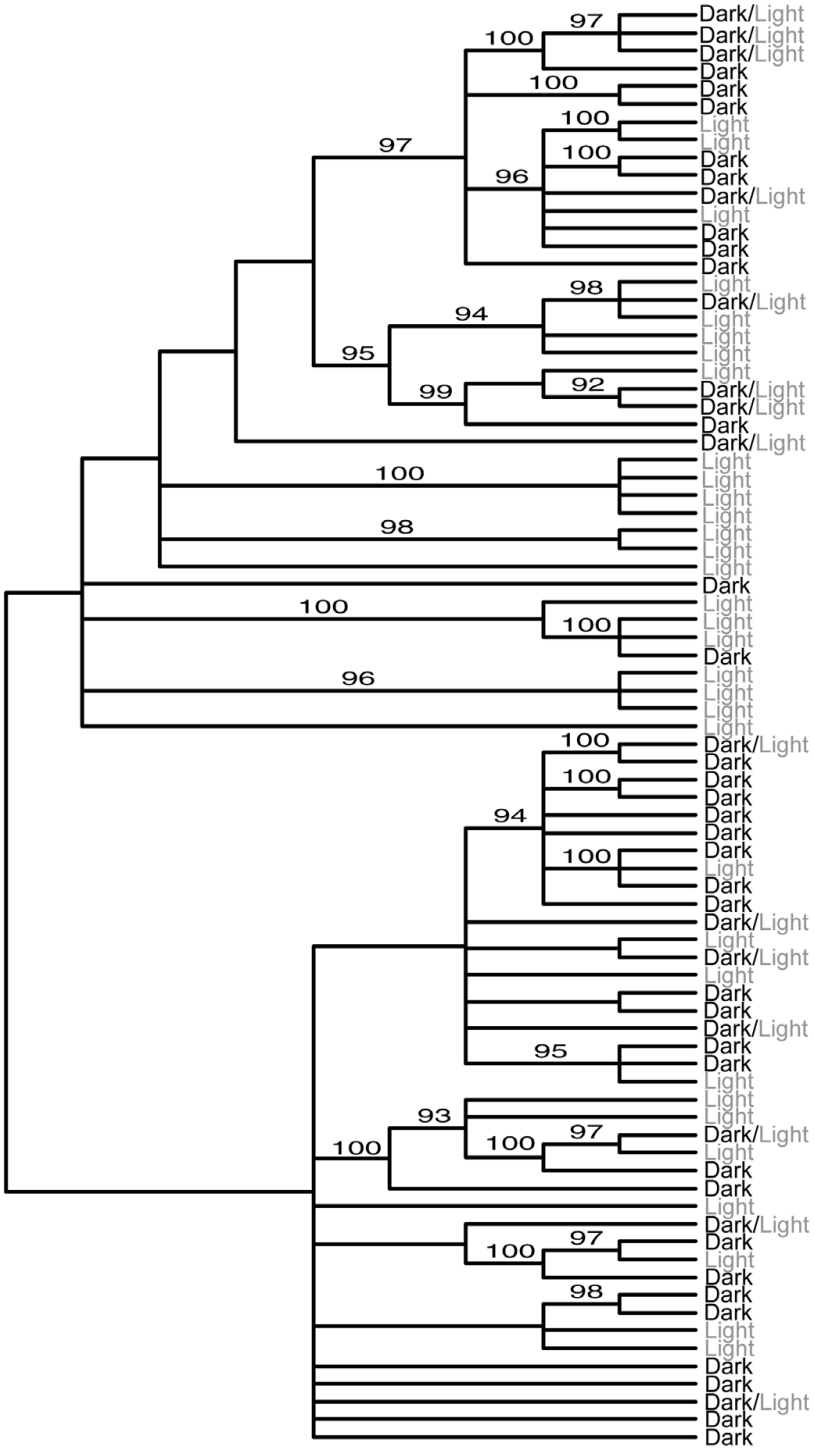
mtDNA Phylogeny. MrBayes tree for combined mtDNA haplotypes. Posterior probabilities greater than 90 are displayed on branches. Statistically supported clades that correspond to sublocalities are listed. Haplotypes are associated with lizards whose dorsal coloration is either light, dark, or both (dark/light).

Sequencing of mitochondrial genes produced 1086 basepairs of *cytb* and 754 basepairs of *nd4,* for a total of 1840 basepairs. We identified 65 haplotypes with 71 polymorphic sites for *cytb*, and 46 haplotypes with 56 polymorphic sites for *nd4*. Combining the two genes resulted in a total of 81 unique mitochondrial haplotypes. We sequenced 701 basepairs of the autosomal gene *Mc1r* with 42 polymorphic sites and 66 haplotypes estimated using the haplotypes considered most likely by PHASE. Haplotype phylogenies for *Mc1r* and the combined mtDNA data are shown in Figures 4 and 5. There is no phylogenetic clustering of color variants and very little substructuring based on locality, although a few small statistically supported clades correspond to groups of individuals collected from the same region. The trees are midpoint rooted, however there is no other possible root that would yield different conclusions.

### Population Genetic Analysis - Overall Molecular Diversity

Data for the entire sample of 127 individuals show a substantial amount of molecular variation (Tables 1 and 2)**.** *Cytb* shows the most variation. Haplotype diversity is extremely high (*h* = .979) with 65 haplotypes. Sequence diversity is also high (π = .007, θ = 0.016). The pairwise mismatch analysis of *cytb* shows a unimodal distribution (*r* = .011, p = .630 (sum of squared deviation tests agreed with *r* in every case and are not reported) and Tajima’s D is significantly negative (D = −1.869, p = .009). *ND4* also shows similarly high molecular diversity (46 haplotypes, *h* = 0.948**,** π = 0.006, θ = 0.014) as well as unimodal distribution of pairwise mismatches (*r* = 0.025, p = 0.110) and a negative Tajima’s D (D = −1.831, p = 0.007).

**Table 1:** Molecular Diversity Summary Statistics.

Locus	bp	N	Variable Sites	Number of Haplotypes	h	π	θ
***cytb***	1086	127	71	65	0.979	0.007	0.016
***ND4***	754	127	56	46	0.948	0.006	0.014
**Combined mtDNA**	1840	127	128	81	0.988	0.006	0.015
***Mc1r*** ** (PHASE)**	701	127	42	66	0.926	0.004	0.010
***Mc1r*** ** (common)**	701	125	42	71	0.898	0.004	0.010

Summary statistics for the two mitochondrial loci and their combined data as well as *Mc1r* using most probable haplotypes (PHASE) and most common haplotypes (common) as described in the text. Included is the number of base pairs sequenced (bp), number of variable sites, number of haplotypes, haplotype diversity (*h*), Nei's θ (π), and Watterson’s θ (θ).

**Table 2:** Mismatch and Neutrality Tests.

Locus	D	P	*r*	P
***cytb***	−1.869	0.009*	0.011	0.630
***ND4***	−1.831	0.007*	0.025	0.110
**Combined mtDNA**	−1.944	0.006*	0.004	0.810
***Mc1r*** ** (PHASE)**	−1.699	0.012*	0.021	0.719
***Mc1r*** ** (common)**	−1.755	0.011*	0.018	0.811

Tajima’s D (D) and raggedness index (*r*) for each locus. Significant P-values are indicated with an asterisk.

**Table 3:** Subsample Molecular Diversity.

mtDNA						
Population	N	VariableSites	Number ofHaplotypes	h	π	θ
**DSC**	17	32	15	0.985	0.004	0.006
**Lava**	110	116	67	0.985	0.006	0.014
**Light**	61	100	47	0.991	0.006	0.014
**Light (No DSC)**	44	81	33	0.985	0.006	0.012
**Dark**	66	100	48	0.987	0.006	0.014
***Mc1r***						
**Population**	**N**	**Variable** **Sites**	**Number of** **Haplotypes**	**h**	**π**	**θ**
**DSC**	34	18	17	0.952	0.005	0.006
**Lava**	220	39	54	0.919	0.004	0.009
**Light**	122	33	42	0.920	0.004	0.009
**Light (No DSC)**	88	30	32	0.904	0.004	0.008
**Dark**	132	31	44	0.921	0.004	0.008

Molecular diversity values for each subsample for mtDNA and *Mc1r*. Number of Individuals (N), number of variable sites, number of haplotypes, haplotype diversity (*h*), nucleotide diversity (π), and Watterson’s θ (θ) are included for each subsample.

**Table 4:** Subsample Mismatch and Neutrality Tests.

mtDNA				
Population	D	P	r	P
**DSC**	−1.392	0.079	0.017	0.830
**Lava**	−1.854	0.008*	0.004	0.800
**Light**	−1.942	0.004*	0.003	0.950
**Light (No DSC)**	−1.694	0.024	0.005	0.920
**Dark**	−1.862	0.009*	0.009	0.340
***Mc1r***				
**Population**	**D**	**P**	**r**	**P**
**DSC**	−0.897	0.196	0.024	0.653
**Lava**	−0.168	0.012*	0.030	0.738
**Light**	−1.615	0.026*	0.019	0.820
**Light (No DSC)**	−1.672	0.018*	0.017	0.870
**Dark**	−1.526	0.030*	0.022	0.690

Tajima’s D (D) and raggedness index (*r*) for each subsample. Significant values are indicated with an asterisk.

**Table 5:** Population Genetic Differentiation.

mtDNA						
Comparison	FST	P	ΦST	P	D_j_	P
**Light vs. Dark**	−0.001	0.577	0.017	0.016*	−0.045	0.561
**Light vs. Dark (No DSC)**	−0.002	0.792	−0.001	0.460	−0.168	0.756
**Lava vs. DSC**	0.014	0.012*	0.125	0.000*	0.964	0.001*
***Mc1r***						
**Comparison**	**FST**	**P**	**ΦST**	**P**	**D_j_**	**P**
**Light vs. Dark**	0.008	0.015*	0.006	0.068	0.079	0.066
**Light vs. Dark (No DSC)**	0.010	0.022*	0.006	0.095	0.076	0.089
**Lava vs. DSC**	0.020	0.017*	0.019	0.011*	0.079	0.166

The combined mitochondrial dataset (*ND4 and cytb)* shows diversity statistics that are consistent with the individual mitochondrial genes (81 haplotypes, *h* = 0.988, π = .006, θ = 0.015) as well as a unimodal distribution of pairwise mismatches (*r* = 0.004, p = 0.81) and significantly negative Tajima’s D (D = −1.944, p = .006). Maximum sequence divergence between any two mitochondrial haplotypes was only 1.25%. Most of the high diversity at the nucleotide level is due to the large number and fairly equal distribution of haplotypes.

As expected, the autosomal gene *Mc1r* shows less variation than mitochondrial loci, yet is still very diverse for a nuclear protein coding locus. Haplotype diversity is extraordinarily high (*h* = .926) with 66 haplotypes in 127 individuals. Sequence diversity is also high (π = 0.004, θ = 0.010). Tests for mismatch distribution show a unimodal distribution (*r* = 0.021, p = .719) and Tajima’s D deviates from neutrality (D = −1.699, p = .012). Maximum sequence divergence between any two *Mc1r* haplotypes was 1.28%. All these values are consistent with those from the mitochondrial loci.

However, because of the high *Mc1r* diversity, PHASE could not assign haplotype identities to all genotypes with high probabilities, and it is possible that the diversity values were inflated because of misassigned haplotypes. In order to be conservative, we reanalyzed the data by examining all possible haplotype designations for each individual with multiple possible genotypes and preferentially choosing the genotype that minimized the probability that PHASE artificially created excess diversity. This was done by preferentially assigning genotypes that involved one of the two most common haplotypes (with frequencies of 0.24 and 0.20 after all genotypes were assigned) even if PHASE estimated higher probabilities for genotypes with rarer haplotypes. If none of the possible genotypes contained one of these common haplotypes, genotypes were chosen if they contained a haplotype that is homozygous in at least one other individual, and therefore is known to exist with certainty. Two individuals were dropped from the analysis because they could not be assigned genotypes under these criteria. Arlequin analysis shows that this reassignment of haplotypes did not significantly alter the diversity of the *Mc1r* sample. There were 71 unique sequences in the 125 individuals (*h* = 0.898) and nucleotide diversity values were similar to the PHASE predicted data set (π = 0.004, θ = 0.010). Pairwise mismatch and Tajima’s D values were also similar (*r* = 0.018, p = 0.809; D = −1.755 p = 0.011). This suggests that the high diversity values for this locus are not due to PHASE artifacts.

The high levels of diversity seen in the overall sample could potentially be due to restricted gene flow between the Desert Studies Center and the Lava collecting sites, or incipient reproductive isolation between light and dark lizards. Therefore we examine population structure based on geography or color in the next section.

### Population Genetic Analysis - Population Structure and Local Diversity

If selection for background matching has resulted in reproductive isolation between light and dark lizards, or if geographic barriers have isolated regions of our study area, population structure statistics may reveal differences among these groups even if there is no phylogenetic signal separating them. Given that mitochondrial genes do not undergo recombination and that separate analysis of the mitochondrial genes shows they have similar values for all diversity and population structure statistics, we will only describe the statistics for the combined mtDNA.

Table 3 and Table 4 show molecular diversity, mismatch distributions, and Tajima’s D test for each defined subsample. When comparing putative subpopulations separated by the soda lake, the lava (and adjacent habitats) population shows molecular diversity that is similar to the Desert Studies Center population. Both geographic samples are similar to the combined sample values for all diversity statistics. Tajima’s D is significantly negative for the Lava population and negative but not significant for the Desert Studies Center population, although the smaller sample size (N = 17) at the Desert Studies Center may be the reason for the lack of statistical significance. Each comparison also shows a unimodal pairwise mismatch distribution. Population structure statistics for mitochondrial loci (Table 5) for this comparison suggest strong population subdivision (ΦST = 0.125, p<0.001; D_j_ = .964, p = 0.001). *Mc1r* does show a weak and statistically significant ΦST (ΦST = 0.019, p = 0.011), however Jost’s D is not statistically significantly different from 0 (D_j = _0.083, p = 0.166). Molecular diversity, mismatch distributions and Tajima’s D statistics are compatible with the overall values, which suggests that population subdivision is not solely responsible for any of the high diversity values for the combined analysis.

When comparing dark and light lizards, population structure analysis of mtDNA indicates weak but statistically significant substructure in mitochondrial loci, but only when the Desert Studies Center individuals are included (ΦST = 0.017, p = 0.016). When the Desert Studies Center animals are removed from the analysis, no significant ΦST or Jost’s D values are found. Although ΦST and Jost’s D values show low levels of differentiation for *Mc1r;* the P-values are just marginally insignificant (ΦST = 0.006 p = 0.068 D_j_ = 0.079, p = .066).

## Discussion

### Phenotypic Plasticity

Our small experiment suggests that adult phenotypic plasticity may not explain the dramatic color variation in our population of side-blotched lizards residing on different substrates. When housed on light substrates, most lizards did not change color, and those that did changed very little. [42] similarly found that *Holbrookia maculata* (Common Lesser Eared Lizard) and *Sceloporus undulatus* (Eastern Fence Lizard) vary in color based on substrate habitat, but show no physiological color change over time when reared on an intermediate colored substrate. Other studies suggest that captive reared hatchlings of other lizard species in similar systems develop to match maternal coloration, not substrate color [42] [9]. Because environmentally induced variation probably cannot explain patterns of dorsal coloration in *U. stansburiana* it is appropriate to consider the role of natural selection for local substrate matching in shaping observed phenotypic variation.

However, our study of adult phenotypic plasticity and the conclusion that color matching is an evolved condition should be considered preliminary. Sample size in our experiment was very small, mostly because of the Federally protected status of our study site. We examined only one possible direction of color change (dark to light) and the ability of these lizards to change to match dark substrate was not tested. In addition, we examined only adults. Color matching may occur during the development of hatchlings. Finally, a lack of phenotypic plasticity is not synonymous with heritability. Accurate estimation of heritability requires very large sample sizes and complex breeding designs to eliminate maternal effects. But it would be possible to conduct a smaller comparison of female/offspring phenotypic similarity.

### Genetic Basis of Color

*Mc1r* did not show any correlation to color based on phylogenetic and population genetic analyses. Rosenblum et al. [3] found that one amino acid substitution in *Mc1r* is highly associated with lighter color variants of Little Striped Whiptail, *Aspidocelis inornata* and [1] found the same to be true in pocket mice, *Chaetodipus intermedius*. However, many other species showing color variation also show no correlation between color and *Mc1r* e.g. [43 44 45 46 ]. Most polymorphic sites in our *Mc1r* sequences are synonymous base changes, and the 5 non-synonymous base changes are found in too few individuals to correlate with color variation. Although we were unable to sequence the entire gene coding region, we were able to include the nucleotide sites known to affect color variation in reptiles [3 44].

### Molecular Diversity

Perhaps the most interesting result of our study is that both mitochondrial DNA and autosomal DNA show an extraordinary amount of diversity in our sample. Haplotype diversity (*h*) and diversity estimates for π and θ are higher in our limited lava field area than seen in some globally sampled data sets in other taxa. Diversity is higher even when comparing *U. stansburiana* to other taxa that show adaptive color variation on lava flows. For instance dark variants of pocket mice, *Chaetodipus intermedius,* on lava fields across Arizona and New Mexico have a π of about.002 for *Mc1r* [24]. θ estimates for combined populations of beach mice are around.005 for *Mc1r* [47], whereas our *Mc1r* θ estimates are double that (θ = .010). Our diversity estimates are comparable to values found in marine fish samples taken over a global scale. For instance, the Pelagic Wahoo, *Acanthocybium solandri* **,** collected from multiple oceans around the world, has high values of genetic diversity (*h* = 0.918, π = .006 and θ = .006) for *cytb* [48] and global samples of the Squirrelfish, *Holocentrus ascensionis,* also showed similar levels of cytb diversity (*h = *.976 and π = .006) [49]. However, we found a diversity values of *h = *0.979, π = .007 and θ = .016 for *cytb* in an area easily small enough to walk across in a day. The high levels of genetic variation in *Uta stansburiana* are even more geographically localized. The Desert Studies Center samples were all collected within an area <5 km across (all but three from an area <.7 km across). Yet the haplotype diversity of this sample is 0.985. We also examined subdivision among the three separated areas within the Lava sample. If we restrict our inference to the south west corner of the lava only, the haplotype diversity for cytb was 0.960 (n = 65), with the highest allele frequency = 0.123 (8 individuals). Yet the two most separated samples were less than 10 km apart, and most samples were much closer. The fact that genetic variation in worldwide populations of highly vagile marine fish is similar to genetic variation of our lizards on a single lava field indicates extraordinary variation.

Reported sequence and haplotype diversities in DNA sequencing studies of lizards have tended to be high (reviewed in [50]). However, because most of these studies have concentrated on phylogeography, local sample sizes have mostly been very small, with resulting poor estimates of diversity. Camargo et al [50] estimate an average per locality sample size of 12.4, and for many of those studies “localities” can refer to a very widespread area. We know of few lizard studies with local sample sizes comparable to ours. Clark et al [51] report haplotype diversities for *Sceloporus woodi* from 3 “localities” of 0.71 (N = 36), 0.69 (N = 39) and 0.76 (n = 29), although even these localities contain samples collected over tens or hundreds of kilometers. Mulcahy et al [52] sampled 4 populations of *Phrynosoma mcalli* for ND4 sequence variation and found haplotype diversities of 0.555, 0.730, 0.762, and 0.942. However the site with the largest h value contained samples separated by over 75 km and surely included separate populations with independent drift effects on allele frequencies. Corso et al [44] studied mc1r variation in two species of *Liolemis* across their entire ranges in Brazil and reported h values of 0.384 (*L. arambarensis*) and 0.813 (*L. occiptalis*).

A few lizard studies with medium to large sample sizes have found high levels of genetic diversity. Rosenblum et al. [17] found mtDNA genetic diversity in *S. undulatus* on the Carrizozo lava flow and White Sands region of New Mexico to be as high as we have found in *U. stansburiana, albeit* over a somewhat larger area. Sequence divergence among haplotypes was even greater. Rosenblum et al [3] found high levels of mc1r variation in several species occupying the same region of New Mexico studied by [17]. It may be that many species in these geologically active area have larger and/or expanding populations, perhaps because of invasion of newly available habitats. We are investigation genetic variation in a large number of lizard and snake species found in the region of the Cima volcanic field to see if other taxa have high diversity and if there are ecological correlates with diversity. Very preliminary results suggest that some species have high diversity while others do not.

The genetic diversity we found in Uta stansburiana is unlikely to be due to any sequencing error or artifacts. In addition to obtaining sequence in both directions, we resequenced individuals if sequence data did not have distinct peaks or showed suspicious base changes. Once all the data were acquired we found no unexpected stop codons, and very few non-synonymous base changes. Furthermore, heterozygote base calling was conservative; a position was deemed heterozygous only if the sequence data showed clear double peaks in both directions.

There are a number of mechanisms that could account for the high levels of current genetic variability. One mechanism directly implied by our analysis is population expansion. Both Tajima’s D values and pairwise mismatch distributions show consistent results in the total sample for both mitochondrial and autosomal DNA. A significantly negative Tajima’s D means that there is an excess of low frequency polymorphisms (rare variation), consistent with population growth or positive selection. The fact that both a mitochondrial and an autosomal gene show similar patterns suggest that population growth is more likely than selection.

### Conclusion

Investigation of this system shows a clear distribution of lighter animals on granite and associated rocks and darker animals on dark, basalt rock. Our tests and related studies suggest that this variation in color is not due to phenotypic plasticity. Although the candidate locus *Mc1r* is highly variable in our samples, it does not correlate with the observed color differences. Still, this variability, along with the extreme variability seen in our mitochondrial DNA, suggests that this species may be undergoing population expansion and may have a large population size in the Cima Lava Field.

Future studies should investigate similar lava field systems of *U. stansburiana* to determine if this diversity is consistent in other locations. The Pisgah Volcano, also in San Bernardino County, is a younger volcano with a population of *U. stansburiana* that varies in color [8]. A comparison of these two sites could show how volcanic age is correlated with genetic variation. Other populations of *U. stansburiana* that do not inhabit lava fields should be investigated as well to ensure this level of variation is not common in this species. Finally other candidate color genes should be sequenced to see if they correlate with different dorsal color variants. This work contributes to our knowledge of adaptive traits on novel habitats, and will hopefully lead to future studies identifying the underlying mechanisms of the color polymorphism associated with habitats with dramatically contrasting substrate colors.

## Supporting Information

Table S1 Collection Data.
(DOC)

Table S2 Polymerase Chain Reaction Recipes.
(DOC)

Table S3 Polymerase Chain Reaction Protocols.
(DOC)

Table S4 PCR and Sequencing Primers.
(DOC)

Table S5 Munsell Geological Rock-Color Chart Color Calls.
(DOC)
