# Supplementary material for: Adaptive Color Polymorphism and Unusually High Local Genetic Diversity in the Side-Blotched Lizard, Uta stansburiana
Source: PLoS One. 2012 Oct 25;7(10):e47694. doi: 10.1371/journal.pone.0047694 (PMC3485026; doi:10.1371/journal.pone.0047694)
Supplement: Table S1 — Collection Data. (DOC) [file pone.0047694.s001.doc]

**Table S1: Collection Data**

| **Lizard ID** | **Lizard Color** | **STV Length (cm)** | **Sex** | **Collection Location** | **GPS North** | **GPS West** | **Elevation (m)** |
| --- | --- | --- | --- | --- | --- | --- | --- |
| 1 | Dark | 4.1 | M | S. Lava | 35.18666 | 115.76699 | 1022 |
| 2 | Dark | 4 | F | S. Lava | 35.18389 | 115.76834 | 1020 |
| 3 | Dark | 4.2 | F | W. Lava | 35.19930 | 115.86356 | 708 |
| 4 | Dark | 3.9 | F | W. Lava | 35.19827 | 115.85777 | 731 |
| 6 | Dark | 3.9 | F | W. Lava | 35.19854 | 115.85045 | 754 |
| 7 | Dark | 5 | M | W. Lava | 35.19519 | 115.84063 | 786 |
| 8 | Dark | 4.3 | M | W. Lava | 35.19435 | 115.84007 | 786 |
| 9 | Dark | 4.8 | M | W. Lava | 35.18813 | 115.84016 | 779 |
| 10 | Dark | 4.3 | M | W. Lava | 35.18929 | 115.84853 | 755 |
| 11 | Dark | 4 | M | W. Lava | 35.19925 | 115.86893 | 680 |
| 12 | Dark | 4 | M | W. Lava | 35.20052 | 115.87173 | 675 |
| 13 | Dark | 4.6 | M | W. Lava | 35.19927 | 115.87161 | 680 |
| 14 | Dark | 4.6 | M | W. Lava | 35.19683 | 115.86925 | 688 |
| 15 | Dark | 4.4 | M | W. Lava | 35.19601 | 115.86894 | 694 |
| 16 | Dark | 4.1 | F | W. Lava | 35.18874 | 115.85643 | 730 |
| 17 | Dark | 4.2 | F | W. Lava | 35.18806 | 115.84843 | 757 |
| 18 | Dark | NA | M | W. Lava | 35.18893 | 115.84326 | 772 |
| 19 | Dark | 4.3 | M | W. Lava | 35.19375 | 115.84240 | 770 |
| 20 | Dark | NA | M | W. Lava | 35.19613 | 115.85579 | 728 |
| 22 | Dark | 4.5 | M | W. Lava | 35.20071 | 115.87244 | 674 |
| 23 | Dark | 4.4 | F | W. Lava | 35.18519 | 115.84745 | 764 |
| 24 | Dark | 4.2 | F | W. Lava | 35.18224 | 115.83325 | 807 |
| 25 | Dark | 4.7 | M | W. Lava | 35.18378 | 115.82997 | 802 |
| 26 | Dark | 4.4 | F | W. Lava | 35.18626 | 115.82051 | 862 |
| 27 | Dark | 4.3 | M | W. Lava | 35.18609 | 115.82127 | 850 |
| 28 | Dark | 4.2 | F | W. Lava | 35.18745 | 115.82337 | 854 |
| 29 | Light | NA | M | DSC | 35.16284 | 116.10754 | 294 |
| 30 | Light | 5.1 | M | DSC | 35.14280 | 116.10395 | 294 |
| 31 | Light | 4.4 | F | DSC | 35.14300 | 116.10327 | 289 |
| 32 | Light | 4.3 | F | DSC | 35.14300 | 116.10321 | 288 |
| 33 | Light | 5 | M | DSC | 35.14261 | 116.10317 | 285 |
| 34 | Light | 4.6 | M | DSC | 35.14141 | 116.10528 | 281 |
| 35 | Light | 2.3 | F | DSC | 35.13995 | 116.10594 | 286 |
| 36 | Light | 4.5 | F | DSC | 35.14013 | 116.10629 | 271 |
| 37 | Light | 5.3 | M | DSC | 35.14007 | 116.10670 | 283 |
| 38 | Light | NA | F | W. Lava | 35.22117 | 115.89020 | 600 |
| 39 | Light | 4.4 | F | W. Lava | 35.22117 | 115.89020 | 600 |
| 40 | Dark | 4.7 | F | S. Lava | 35.17071 | 115.82032 | 852 |
| 41 | Dark | 4.4 | F | S. Lava | 35.17103 | 115.81965 | 848 |
| 42 | Dark | 4.6 | M | S. Lava | 35.17377 | 115.81586 | 864 |
| 43 | Dark | 4.5 | M | S. Lava | 35.17334 | 115.81672 | 857 |
| 44 | Dark | NA | M | S. Lava | 35.17303 | 115.81738 | 853 |
| 45 | Dark | 4.8 | M | S. Lava | 35.16899 | 115.82136 | 845 |
| 46 | Light | NA | M | W. Lava | 35.21750 | 115.89040 | 609 |
| 47 | Light | 4.4 | M | W. Lava | 35.21753 | 115.89039 | 613 |
| 48 | Dark | 4.6 | M | S. Lava | 35.18483 | 115.76699 | 1019 |
| 49 | Dark | 4.6 | M | S. Lava | 35.18523 | 115.76686 | 1019 |
| 50 | Dark | 4.4 | F | E. Lava | 35.21288 | 115.75351 | 1072 |
| 51 | Dark | 4.5 | F | E. Lava | 35.21624 | 115.75189 | 1094 |
| 52 | Dark | NA | F | E. Lava | 35.21620 | 115.75225 | 1093 |
| 53 | Dark | 4.6 | F | E. Lava | 35.21541 | 115.75241 | 1082 |
| 54 | Dark | NA | F | E. Lava | 35.21485 | 115.75280 | 1084 |
| 55 | Dark | 5 | M | E. Lava | 35.21479 | 115.75283 | 1085 |
| 56 | Dark | 4.9 | M | E. Lava | 35.21412 | 115.75315 | 1080 |
| 57 | Dark | 4.5 | M | E. Lava | 35.22199 | 115.73843 | 1146 |
| 59 | Light | 4.3 | F | W. Lava | 35.20112 | 115.87302 | 671 |
| 60 | Dark | 4.5 | M | W. Lava | 35.21031 | 115.87733 | 647 |
| 61 | Dark | NA | M | W. Lava | 35.20933 | 115.87958 | 633 |
| 62 | Dark | 4.2 | F | W. Lava | 35.21029 | 115.88094 | 630 |
| 63 | Dark | 4.3 | M | W. Lava | 35.24901 | 115.88827 | 612 |
| 64 | Dark | 4.5 | M | W. Lava | 35.24919 | 115.88850 | 614 |
| 65 | Light | NA | M | W. Lava | 35.24899 | 115.88928 | 620 |
| 66 | Light | 4.8 | F | W. Lava | 35.24888 | 115.88941 | 625 |
| 67 | Light | 4.5 | F | W. Lava | 35.24845 | 115.88960 | 616 |
| 68 | Light | 4.6 | F | W. Lava | 35.24679 | 115.88895 | 612 |
| 69 | Dark | 4.4 | F | W. Lava | 35.20832 | 115.88481 | 639 |
| 70 | Light | 4.6 | M | W. Lava | 35.20883 | 115.88577 | 643 |
| 71 | Light | 4.5 | M | W. Lava | 35.20848 | 115.88747 | 647 |
| 72 | Light | 4.3 | F | DSC | 35.13663 | 116.10733 | 525 |
| 73 | Light | 4.5 | M | DSC | 35.13835 | 116.10820 | 285 |
| 74 | Light | 4.6 | M | DSC | 35.13882 | 116.10714 | 276 |
| 78 | Light | 4.2 | F | DSC | 35.149727 | 116.1068 | 290 |
| 79 | Light | 4.9 | M | DSC | 35.149584 | 116.1064 | 286 |
| 80 | Light | 3.8 | M | DSC | 35.178772 | 116.1139 | 304 |
| 81 | Light | 3.3 | M | E. Lava | 35.219285 | 115.7679 | 1042 |
| 82 | Dark | 4 | M | E. Lava | 35.220301 | 115.7750 | 1047 |
| 83 | Dark | 3.7 | M | E. Lava | 35.220303 | 115.7750 | 1047 |
| 84 | Dark | 4.8 | M | E. Lava | 35.221665 | 115.7784 | 1060 |
| 85 | Light | 3.9 | F | E. Lava | 35.221244 | 115.7789 | 1062 |
| 86 | Light | 3.7 | F | E. Lava | 35.221119 | 115.7800 | 1072 |
| 87 | Dark | 3.6 | F | E. Lava | 35.221119 | 115.7800 | 1071 |
| 88 | Dark | 3.4 | M | E. Lava | 35.221221 | 115.7870 | 1034 |
| 89 | Light | 4.8 | M | E. Lava | 35.222245 | 115.7879 | 1031 |
| 90 | Light | 3.1 | F | E. Lava | 35.222491 | 115.7879 | 1041 |
| 91 | Dark | 3.4 | F | E. Lava | 35.222662 | 115.7877 | 1041 |
| 92 | Light | 5.1 | F | E. Lava | 35.223628 | 115.7896 | 1033 |
| 93 | Dark | 3.9 | M | E. Lava | 35.22362 | 115.7896 | 1032 |
| 94 | Light | 4.1 | F | E. Lava | 35.223769 | 115.7897 | 1032 |
| 95 | Light | 3.5 | M | E. Lava | 35.22377 | 115.7897 | 1032 |
| 96 | Light | 4 | M | E. Lava | 35.224238 | 115.7919 | 1023 |
| 97 | Dark | 3.9 | M | E. Lava | 35.223338 | 115.7909 | 1028 |
| 98 | Light | 4.5 | M | E. Lava | 35.221942 | 115.7892 | 1033 |
| 99 | Light | 3.7 | F | E. Lava | 35.222465 | 115.7901 | 1026 |
| 100 | Light | 3.1 | F | E. Lava | 35.221647 | 115.7890 | 1028 |
| 101 | Dark | 3.7 | M | E. Lava | 35.216671 | 115.7816 | 1058 |
| 102 | Light | 3.6 | M | E. Lava | 35.216824 | 115.7794 | 1053 |
| 103 | Light | 4.6 | M | W. Lava | 35.200535 | 115.8776 | 690 |
| 104 | Light | 3.8 | M | W. Lava | 35.201636 | 115.8782 | 687 |
| 105 | Dark | 3.6 | M | W. Lava | 35.202096 | 115.8776 | 697 |
| 106 | Light | 4.1 | F | W. Lava | 35.220142 | 115.8915 | 616 |
| 107 | Dark | 4.2 | M | W. Lava | 35.220524 | 115.8919 | 619 |
| 108 | Light | 3 | F | W. Lava | 35.220527 | 115.8919 | 618 |
| 109 | Light | 3.6 | M | W. Lava | 35.220534 | 115.8919 | 618 |
| 110 | Light | 3.7 | F | W. Lava | 35.219962 | 115.8912 | 604 |
| 111 | Light | 4.1 | M | W. Lava | 35.219068 | 115.8903 | 607 |
| 112 | Light | NA | N/A | W. Lava | 35.218487 | 115.8895 | 610 |
| 113 | Light | 3.6 | F | W. Lava | 35.217918 | 115.8881 | 615 |
| 114 | Light | 3.9 | M | W. Lava | 35.217917 | 115.8881 | 615 |
| 115 | Light | 3.5 | F | W. Lava | 35.217852 | 115.8880 | 616 |
| 116 | Dark | 4.4 | M | W. Lava | 35.217852 | 115.8880 | 616 |
| 117 | Light | 3.5 | F | W. Lava | 35.217584 | 115.8874 | 615 |
| 118 | Light | 4.8 | M | W. Lava | 35.210237 | 115.8854 | 640 |
| 119 | Light | 3.9 | F | W. Lava | 35.208477 | 115.8872 | 654 |
| 120 | Light | 4.3 | M | W. Lava | 35.209206 | 115.8871 | 651 |
| 121 | Light | NA | M | W. Lava | 35.20564 | 115.8859 | 652 |
| 122 | Dark | 4.6 | F | W. Lava | 35.206625 | 115.8853 | 643 |
| 123 | Light | 3.5 | F | W. Lava | 35.206641 | 115.8853 | 648 |
| 124 | Light | 3.5 | F | W. Lava | 35.208538 | 115.8847 | 647 |
| 125 | Light | 4.1 | F | W. Lava | 35.208485 | 115.8845 | 647 |
| 126 | Light | 3.6 | F | W. Lava | 35.247613 | 115.8902 | 602 |
| 127 | Dark | NA | M | E. Lava | 35.21687 | 115.75271 | 1089 |
| 128 | Dark | NA | F | E. Lava | 35.21662 | 115.75373 | 1086 |
| 132 | Dark | NA | F | W. Lava | 35.31639 | 115.55175 | 1536 |
| 133 | Dark | NA | F | W. Lava | 35.31655 | 115.54985 | 1524 |
| 134 | Dark | NA | F | W. Lava | 35.3167 | 115.54630 | 1519 |
| 135 | Light | NA | M | DSC | 35.13964 | 116.10372 | 290 |
| 136 | Light | NA | F | DSC | 35.13964 | 116.10372 | 290 |
