# Supplementary material for: Adaptive Color Polymorphism and Unusually High Local Genetic Diversity in the Side-Blotched Lizard, Uta stansburiana
Source: PLoS One. 2012 Oct 25;7(10):e47694. doi: 10.1371/journal.pone.0047694 (PMC3485026; doi:10.1371/journal.pone.0047694)
Supplement: Table S2 — Polymerase Chain Reaction Recipes. (DOC) [file pone.0047694.s002.doc]

**Table S2: Polymerase Chain Reaction Recipes**

| ***Mc1r*** |  |  |
| --- | --- | --- |
| **Stock Reagents** | **Amount (Per RXN)** | **Final concentration** |
| Water | 15 μl | - |
| Dream Taq Buffer 10X (20 mM MgCl2) | 2.5 μl | 1X, 2 mM |
| Primer 1 (10 μM) | 1.6 μl | 0.64 μM |
| Primer 2 (10 μM) | 1.6 μl | 0.64 μM |
| dNTPs (10 mM) | 2.6 μl | 1.04 mM |
| Fermentas Dream Taq Polymerase (5 U/μl) | 0.2 μl | 1 U |
| Template DNA (50 ng/μl) | 1.5 μl | 3 ng/μl |
| **Total** | **25 μl** |  |
|  |  |  |
| ***cytb*** |  |  |
| **Stock Reagents** | **Amount (Per RXN)** | **Final concentration** |
| Water | 11.6 μl | - |
| Amplitaq 10 X Buffer II | 2 μl | 1X |
| Primer 1 (10 μM) | 1 μl | 0.5 μM |
| Primer 2 (10 μM) | 1 μl | 0.5 μM |
| dNTPs (10 mM) | 0.6 μl | 0.3 mM |
| MgCl2 (25 mM) | 2.4 μl | 3 mM |
| Fermentas Dream Taq Polymerase (5 U/μl) | 0.2 μl | 1 U |
| Template DNA (50 ng/μl) | 1.2 μl | 3 ng/μl |
| **Total** | **20 μl** |  |
|  |  |  |
| ***ND4*** |  |  |
| **Stock Reagents** | **Amount (Per RXN)** | **Final concentration** |
| Water | 12.5 μl | - |
| Dream Taq Buffer 10X (20 mM MgCl2) | 2.1 μl | 1.05X, 2.1 mM |
| Primer 1 (10 μM) | 1 μl | 0.5 μM |
| Primer 2 (10 μM) | 1 μl | 0.5 μM |
| dNTPs (10 mM) | 2 μl | 1 mM |
| Fermentas Dream Taq Polymerase (5 U/μl) | 0.15 μl | .75 U |
| Template DNA (50 ng/μl) | 1.25 μl | 3 ng/μl |
| **Total** | **20 μl** |  |
