# Supplementary material for: Adaptive Color Polymorphism and Unusually High Local Genetic Diversity in the Side-Blotched Lizard, Uta stansburiana
Source: PLoS One. 2012 Oct 25;7(10):e47694. doi: 10.1371/journal.pone.0047694 (PMC3485026; doi:10.1371/journal.pone.0047694)
Supplement: Table S3 — Polymerase Chain Reaction Protocols. (DOC) [file pone.0047694.s003.doc]

**Table S3: Polymerase Chain Reaction Protocols**

| ***Mc1r*** |  |  |  |
| --- | --- | --- | --- |
| **Step** | **Temperature ˚C** | **Time** | **Number of Cycles** |
| Initial Denaturation | 94˚ | 4 min | 1 |
| Denaturation | 94˚ | 30 sec | 25 |
| Annealing | 67˚ | 35 sec |
| Extension | 72˚ | 50 sec |
| Final Extension | 72˚ | 7 min | 1 |
| Refrigeration | 10˚ | ∞ |  |
|  |  |  |  |
| ***cytb*** |  |  |  |
| **Step** | **Temperature ˚C** | **Time** | **Number of Cycles** |
| Initial Denaturation | 94˚ | 4 min | 1 |
| Denaturation | 94˚ | 30 sec | 25 |
| Annealing | 50˚ | 35 sec |
| Extension | 72˚ | 50 sec |
| Final Extension | 72˚ | 7 min | 1 |
| Refrigeration | 10˚ | ∞ |  |
|  |  |  |  |
| ***ND4*** |  |  |  |
| **Step** | **Temperature ˚C** | **Time** | **Number of Cycles** |
| Initial Denaturation | 94˚ | 4 min | 1 |
| Denaturation | 94˚ | 30 sec | 25 |
| Annealing | 55˚ | 35 sec |
| Extension | 72˚ | 50 sec |
| Final Extension | 72˚ | 7 min | 1 |
| Refrigeration | 10˚ | ∞ |  |
