# Supplementary material for: Adaptive Color Polymorphism and Unusually High Local Genetic Diversity in the Side-Blotched Lizard, Uta stansburiana
Source: PLoS One. 2012 Oct 25;7(10):e47694. doi: 10.1371/journal.pone.0047694 (PMC3485026; doi:10.1371/journal.pone.0047694)
Supplement: Table S4 — PCR and Sequencing Primers. (DOC) [file pone.0047694.s004.doc]

**Table S4: PCR and Sequencing Primers**

| **Locus** | **PCR Primer 1** | **PCR Primer 2** |
| --- | --- | --- |
| ***Mc1r*** | CAGCAARCCCACAGGTGAG  (1) | TGGYTCTCTGGCAGATGATG  (1) |
| ***cytb*** | CCACCGTTGTTATTCAACTAC  (2) | GGTTTACAAGACCAATGCTTT  (2) |
| ***ND4*** | CACCTATGACTACCAAAAGCTCATGTAGAA  (3). | CATTACTTTTACTTGGATTTGCACCA  (3). |

| **Locus** | **Forward Sequencing Primer** | **Reverse Sequencing Primer** |
| --- | --- | --- |
| ***Mc1r*** | GCCCATCAATGTGACCAAC  GATGACGGTTCCATCACCTC | TCAAAGTCCTCCTGAGCT  (1) |
| ***cytb*** | ACTTTGGCTCTCTTTTAGGAC | ATGATTGAGGCTAGTTGTCCG |
| ***ND4*** | ACTAAAACTTGGGGGATATGG | GCAGTTCTTGGTGTTCAAAAC |

References

1. Rosenblum EB, Hoekstra HE, Nachman MW (2004) Adaptive reptile color variation and the evolution of the *Mc1r* gene. Evolution 58:1794–1808.

2. Corl A, Davis AR, Kuchta SR, Comendant T, Sinervo B (2010) Alternative mating strategies and the evolution of sexual size dimorphism in the side-blotched lizard, *Uta stansburiana*: a population-level comparative analysis. Evolution **64**: 79–96.

3. Arevalo, E, Davis SK, Sites JW (1994) Mitochondrial DNA sequence divergence and phylogenetic relationships among eight chromosome races of the *Sceloporus grammicus* complex (Phrynosomatidae) in central Mexico. Syst. Biol. **43**: 387–418.
