# Supplementary material for: Adaptive Color Polymorphism and Unusually High Local Genetic Diversity in the Side-Blotched Lizard, Uta stansburiana
Source: PLoS One. 2012 Oct 25;7(10):e47694. doi: 10.1371/journal.pone.0047694 (PMC3485026; doi:10.1371/journal.pone.0047694)
Supplement: Table S5 — Munsell Geological Rock-Color Chart Color Calls. (DOC) [file pone.0047694.s005.doc]

**Table S5: Munsell Geological Rock-Color Chart Color Calls.**

Lizard and substrate colors as determined with the Munsell Rock Chart. Olive black (OB) and olive gray (OG) are associated with dark lizards whereas dark yellowish brown (DYB) and light olive gray (LOG) are associated with light lizards. In some cases the primary dorsal colors fit in between two colors and were assigned two colors. In terms of substrates, brownish gray (BG) is associated with dark basalt rock, whereas medium gray (MG) is associated with lighter granite rocks. Yellowish brown (YB) is associated with soil and tree bark and is also considered light. A lizard is considered to be found matching its substrate if both lizard color class and its substrate color class are the same. Lizards that did not match their substrate are shown in bold. Exceptions to this rule are lizards that had a secondary color that allowed them to match substrates. This generally occurred in lizards that were dark, yet had light spots.

| **Lizard ID** | **Lizard Color** | **Substrate Color** | **Lizard Color** | **Substrate Color** | **Dorsal**  **Temperature (C°)** |
| --- | --- | --- | --- | --- | --- |
|  | **Class** | **Class** |  |  |  |
| 1 | Dark | Dark | OB | BG | 30.56 |
| 2 | Dark | Dark | OG | BG | 32.44 |
| 3 | Dark | Dark | OG | BG | 29.00 |
| 4 | Dark | Dark | OG | BG | 29.11 |
| 6 | Dark | Dark | OG | BG | 30.33 |
| 7 | Dark | Dark | OB | BG | 32.22 |
| 8 | Dark | Dark | OB | BG | 32.06 |
| 9 | Dark | Dark | OB | BG | 34.00 |
| 10 | Dark | Dark | OB | BG | 32.11 |
| 11 | Dark | Dark | OB | BG | 31.28 |
| 12 | Dark | Dark | OB | BG | 33.50 |
| 13 | Dark | Light | OB - DYB | MG | 30.50 |
| **14** | **Dark** | **Light** | **OB** | **MG** | **30.00** |
| 15 | Dark | Dark | OB | BG | 32.22 |
| 16 | Dark | Dark | OG | BG | 34.72 |
| 17 | Dark | Dark | OG | BG | 35.44 |
| 18 | Dark | Dark | OB | BG | 34.44 |
| 19 | Dark | Dark | OB | BG | 31.50 |
| 20 | Dark | Dark | OB | BG | 31.17 |
| 22 | Dark | Dark | OB | BG | 31.00 |
| 23 | Dark | Light | OG -DYB | MG | 32.67 |
| 24 | Dark | Dark | OG | BG | 36.72 |
| 25 | Dark | Dark | OB | BG | 37.94 |
| 26 | Dark | Dark | OG | BG | 35.72 |
| 27 | Dark | Dark | OB | BG | 37.83 |
| 28 | Dark | Dark | OG | BG | 35.78 |
| 29 | Light | Light | DYB | MG | NA |
| 30 | Light | Light | DYB | MG | 35.83 |
| 31 | Light | Light | LOG | MG | 35.00 |
| 32 | Light | Light | LOG | MG | 34.67 |
| 33 | Light | Light | DYB | MG | 34.22 |
| 34 | Light | Light | DYB | MG | 34.00 |
| 35 | Light | Light | LOG | MG | NA |
| 36 | Light | Light | LOG | MG | NA |
| 37 | Light | Light | DYB | MG | 32.83 |
| 38 | Light | Light | LOG | MG | NA |
| 39 | Light | Light | LOG | YB | 38.89 |
| 40 | Dark | Dark | OG | BG | 38.89 |
| 41 | Dark | Dark | OG | BG | 38.28 |
| 42 | Dark | Dark | OB | BG | 37.22 |
| 43 | Dark | Dark | OB | BG | 35.89 |
| 44 | Dark | Dark | OB- OG | BG | NA |
| 45 | Dark | Dark | OB | BG | 34.72 |
| 46 | Light | Light | DYB - OG | MG | NA |
| 47 | Light | Light | DYB | MG | 33.67 |
| 48 | Dark | Dark | OB | BG | 31.11 |
| 49 | Dark | Dark | OB - OG | BG | 32.50 |
| 50 | Dark | Dark | OG | BG | 29.94 |
| 51 | Dark | Dark | OG | BG | 32.11 |
| 52 | Dark | Dark | OG - DYB | BG | NA |
| 53 | Dark | Dark | OG | BG | NA |
| 54 | Dark | Dark | OG - DYB | BG | NA |
| 55 | Dark | Dark | OB - OG | BG | 32.56 |
| 56 | Dark | Dark | OB | BG | 34.50 |
| 57 | Dark | Dark | OB - OG | BG | 35.67 |
| **59** | **Light** | **Dark** | **LOG - OG** | **BG** | 36.72 |
| 60 | Dark | Dark | OB | BG | 36.00 |
| 61 | Dark | Light | OB - OG | MG | 32.17 |
| 62 | Dark | Dark | OG - DYB | BG | 33.11 |
| **63** | **Dark** | **Light** | **OB** | **MG** | **33.67** |
| **64** | **Dark** | **Light** | **OB** | **MG** | **34.00** |
| 65 | Light | Light | DYB - OG | MG | NA |
| 66 | Light | Light | LOG | MG | 29.72 |
| 67 | Light | Light | LOG | MG | 30.22 |
| 68 | Light | Light | LOG | MG | 31.61 |
| **69** | **Dark** | **Light** | **OG** | **MG** | **32.11** |
| 70 | Light | Light | DYB | MG | 37.33 |
| 71 | Light | Light | DYB | MG | 36.89 |
| 72 | Light | Light | LOG | MG | 34.06 |
| 73 | Light | Light | DYB | MG | 33.61 |
| 74 | Light | Light | DYB | MG | 34.33 |
| 78 | Light | Light | LOG | MG | 26.11 |
| 79 | Light | Light | DYB | YB | 26.11 |
| 80 | Light | Light | DYB | MG | 26.11 |
| 81 | Light | Light | DYB - OG | YB | 19.44 |
| 82 | Dark | Light | OB - DYB | MG | 23.78 |
| 83 | Dark | Light | OB - DYB | MG | 23.56 |
| 84 | Dark | Light | OB - DYB | MG | 27.22 |
| 85 | Light | Light | LOG | MG | 22.22 |
| 86 | Light | Light | LOG | MG | 22.22 |
| 87 | Dark | Light | OG - LOG | MG | 22.78 |
| 88 | Dark | Dark | OB | BG | 23.33 |
| 89 | Light | Light | DYB | MG | 21.89 |
| 90 | Light | Light | LOG | MG | 27.22 |
| **91** | **Dark** | **Light** | **OG** | **MG** | **24.44** |
| 92 | Light | Light | LOG - OG | MG | 22.39 |
| **93** | **Dark** | **Light** | **OB** | **MG** | 27.56 |
| 94 | Light | Light | LOG | MG | 21.89 |
| 95 | Light | Light | DYB | MG | 22.83 |
| 96 | Light | Light | DYB | MG | 22.22 |
| 97 | Dark | Light | OB - DYB | MG | 25.00 |
| 98 | Light | Light | DYB | MG | 26.06 |
| **99** | **Light** | **Dark** | **LOG** | **YB** | **25.94** |
| 100 | Light | Light | LOG - OG | MG | 24.44 |
| **101** | **Dark** | **Light** | **OB** | **MG** | **24.44** |
| 102 | Light | Light | DYB | MG | 23.61 |
| 103 | Light | Light | DYB | MG | 22.78 |
| 104 | Light | Light | DYB - OG | MG | 27.22 |
| 105 | Dark | Light | OB - DYB | MG | 29.44 |
| 106 | Light | Light | LOG | MG | 28.33 |
| 107 | Dark | Light | OB - OG | MG | 28.89 |
| 108 | Light | Light | LOG - OG | MG | 30.00 |
| 109 | Light | Light | DYB | MG | 32.78 |
| 110 | Light | Light | LOG | MG | 28.33 |
| 111 | Light | Light | DYB | MG | 30.56 |
| 112 | Light | Light | DYB - OG | MG | NA |
| 113 | Light | Light | LOG | MG | 30.56 |
| 114 | Light | Light | DYB | YB | 29.44 |
| 115 | Light | Light | LOG - OG | MG | 29.44 |
| 116 | Dark | Dark | OB | YB | 26.67 |
| 117 | Light | Light | LOG | MG | 27.78 |
| 118 | Light | Light | DYB | MG | 28.06 |
| 119 | Light | Light | LOG | MG | 26.11 |
| 120 | Light | Light | DYB | MG | 27.50 |
| 121 | Light | Light | DYB | MG | 28.00 |
| 122 | Dark | Light | OG - LOG | MG | 27.78 |
| 123 | Light | Light | LOG - OG | MG | 27.22 |
| 124 | Light | Light | LOG - OG | MG | 28.00 |
| 125 | Light | Light | LOG | MG | 26.33 |
| 126 | Light | Light | LOG | MG | 25.00 |
| 127 | Dark | Dark | OB | YB | 32.22 |
| 128 | Dark | Dark | OG | YB | 31.67 |
| 132 | Dark | Light | OG - LOG | MG | 30.56 |
| **133** | **Dark** | **Light** | **OG** | **MG** | **28.89** |
| 134 | Dark | Light | OG - LOG | MG | 30.00 |
| 135 | Light | Light | DYB | MG | 29.44 |
| 136 | Light | Light | LOG | MG | 32.22 |
